# Supplementary material for: Compression hosiery to avoid post-thrombotic syndrome (CHAPS) protocol for a randomised controlled trial (ISRCTN73041168)
Source: BMJ Open. 2021 Apr 12;11(4):e044285. doi: 10.1136/bmjopen-2020-044285 (PMC8048019; doi:10.1136/bmjopen-2020-044285)
Supplement: Supplementary data [file bmjopen-2020-044285supp002.pdf]

CHAPS Trial Commitees

Trial Steering Committee

Bold = independent member

**Dr**	**Peter**	**MacCallum**	**Senior Lecturer in Haematology**	**Chair**
**Dr**	**Susie**	**Shapiro**	**Consultant Haematologist**	**Member**
**Mr**	**Isaac**	**Nyameke**	**Consultant Vascular Surgeon**	**Member**
**Dr**	**Stephen**	**Gerry**	**Senior medical statistician and NIHR doctoral research fellow**	**Member**
Professor	Alun	Davies	Professor of Vascular Surgery	Member
**Mr**	**Andrew**	**Steptowe**	**PPI member**	**Public Observer**
**Mr**	**David**	**Brae**	**PPI member**	**Public Observer**
Mr	Ankur	Thapar	Co-applicant	Observer
Professor	John	Norrie	Senior Statistician	Observer

BMJ Publishing Group Limited (BMJ) disclaims all liability and responsibility arising from any reliance placed on this supplemental material which has been supplied by the author(s)

Data monitoring committee

Bold = independent member

**Mr**	**Richard**	**Bulbulia**	**Consultant Vascular Surgeon**	**Chair**
**Dr**	**Natalie**	**Staplin**	**Senior Statistician – Renal Studies Group**	**Member**
**Mr**	**Richard**	**Haynes**	**Associate Professor**	**Member**
**Mr**	**Keith**	**Poskitt**	**Consultant Vascular and General Surgeon**	**Member**
Mr	Imad	Adamestam	Trial Statistician	
