# Supplementary material for: Compression hosiery to avoid post-thrombotic syndrome (CHAPS) protocol for a randomised controlled trial (ISRCTN73041168)
Source: BMJ Open. 2021 Apr 12;11(4):e044285. doi: 10.1136/bmjopen-2020-044285 (PMC8048019; doi:10.1136/bmjopen-2020-044285)
Supplement: Supplementary data [file bmjopen-2020-044285supp003.pdf]

Delete this line, then print on CHAPS Hospital/Trust headed pape

CHAPS

Compression Hosiery to Avoid Post-Thrombotic Syndrome

(CHAPS)

ISRCTN: 73041168

HRA/REC Reference: 19/LO/1585

IRAS 263041

Sponsor Reference: 19CX5434

Acknowledgement

This project is funded by a National Institute for Health Research, Health Technology Assessment programme grant, which is funded by the National Institute for Health Research (NIHR)

Disclaimer

The views expressed in this publication are those of the author(s) and not necessarily those of the MRC, NHS, NIHR or the Department of Health.

Delete this line, then print on Hospital/Trust headed paper

Site ID:	Initials:
Participant Trial ID:	Principal Investigator Name:

Compression Hosiery to Avoid Post-thrombotic Syndrome (CHAPS)

IRAS 263041

PATIENT CONSENT FORM

Please initial box

1. I confirm that I have read and understand the information sheet dated 25/06/2020 (Version 3.0) for the above study and have had the opportunity to ask questions which have been answered fully.

2. I understand that my participation is voluntary and that I am free to leave the study at any time without my medical care or legal rights being affected.

3. I understand that relevant sections of my medical records may be looked at by authorised individuals from the research team, from regulatory bodies, from the study Sponsor, or from the NHS Trust in order to check that the study is being carried out correctly. I give permission, provided that strict confidentiality is maintained, for these bodies to have access to my medical records for the above study.

4. I understand that my mobile phone number and email address will be stored until the end of the study securely by the University of Edinburgh and used to send weekly text message reminders to you until your participation on the study ends

5. I understand that my pseudonymised data will be transferred to the University Of Granada for the analysis.

6. I agree to my data being entered onto a secure database held at the University of Edinburgh, in accordance with the Data Protection Act 2018.

7. I agree to my GP, or any other doctor treating me, being notified of my participation in this study. I agree to my GP being involved in the study, including any necessary exchange of information about me between my GP and the research team.

Thapar A, *et al. BMJ Open* 2021; 11:e044285. doi: 10.1136/bmjopen-2020-044285

8. If during the study my clinical care team determine that I have lost capacity to provide informed consent, I will be withdrawn from the study and any identifiable data collected with consent would be retained and used in the study. ☐

9. I agree to take part in the CHAPS study. ☐

Optional consent section (please initial the appropriate box)

10. I give/do not give consent for information collected about me to be used to support other research in the future, including those outside of the EEA.

Give consent ☐ Do not give consent ☐

11. I give/do not give consent for my data may be linked with appropriate national databases, including Hospital Episode Statistics (HES), and the National Vascular Database as well as for longer term follow-up in the event the trial is extended

Give consent ☐ Do not give consent ☐

12. I give/do not give consent to be contacted in the future with regards to this study, should the study be extended.

Give consent ☐ Do not give consent ☐

Full Name of Participant	Date	Signature
Name of Person Taking Consent	Date	Signature

(1 copy for participant; 1 copy for the patient’s medical notes, 1 copy for the site file)

Thapar A, *et al. BMJ Open* 2021; 11:e044285. doi: 10.1136/bmjopen-2020-044285
