# Supplementary material for: Compression hosiery to avoid post-thrombotic syndrome (CHAPS) protocol for a randomised controlled trial (ISRCTN73041168)
Source: BMJ Open. 2021 Apr 12;11(4):e044285. doi: 10.1136/bmjopen-2020-044285 (PMC8048019; doi:10.1136/bmjopen-2020-044285)
Supplement: Author's manuscript [file bmjopen-2020-044285.draft_revisions.pdf]

BMJ Open

BMJ Open is committed to open peer review. As part of this commitment we make the peer review history of every article we publish publicly available.

When an article is published we post the peer reviewers' comments and the authors' responses online. We also post the versions of the paper that were used during peer review. These are the versions that the peer review comments apply to.

The versions of the paper that follow are the versions that were submitted during the peer review process. They are not the versions of record or the final published versions. They should not be cited or distributed as the published version of this manuscript.

BMJ Open is an open access journal and the full, final, typeset and author-corrected version of record of the manuscript is available on our site with no access controls, subscription charges or pay-per-view fees (http://bmjopen.bmj.com).

If you have any questions on BMJ Open's open peer review process please email info.bmjopen@bmj.com

BMJ Open

Compression Hosiery to Avoid Post-Thrombotic Syndrome (CHAPS)
Protocol for a Randomised Controlled Trial
[ISRCTN73041168]

Journal:	*BMJ Open*
Manuscript ID	bmjopen-2020-044285
Article Type:	Protocol
Date Submitted by the Author:	28-Aug-2020
Complete List of Authors:	Thapar, Ankur; Imperial College London, Academic Section of Vascular Surgery<br>Lawton, Rebecca; Imperial College London, Academic Section of Vascular Surgery<br>Burgess, Laura; Imperial College London, Academic Section of Vascular Surgery<br>Shalhoub, Joseph; Imperial College London, Section of Vascular Surgery<br>Bradbury, Andrew; University of Birmingham, Institute of Cardiovascular Sciences<br>Cullum, Nicky; The University of Manchester, School of Nursing, Midwifery & Social Work<br>Epstein, David; University of Granada, Faculty of Economic and Business Sciences<br>Gohel, Manjit; Addenbrooke's Hospital, Department of Vascular Surgery<br>Horne, Robert; University College London<br>Hunt, Beverley; Guy's & St Thomas' Foundation Trust, Dept of Haematology<br>Norrie, John; Edinburgh Clinical Trials Unit, University of Edinburgh No. 9, Bioquarter<br>Davies, AH; Imperial College London, Vascular
Keywords:	Vascular medicine < INTERNAL MEDICINE, Anticoagulation < HAEMATOLOGY, Thromboembolism < CARDIOLOGY

SCHOLARONE™
Manuscripts

I, the Submitting Author has the right to grant and does grant on behalf of all authors of the Work (as defined in the below author licence), an exclusive licence and/or a non-exclusive licence for contributions from authors who are: i) UK Crown employees; ii) where BMJ has agreed a CC-BY licence shall apply, and/or iii) in accordance with the terms applicable for US Federal Government officers or employees acting as part of their official duties; on a worldwide, perpetual, irrevocable, royalty-free basis to BMJ Publishing Group Ltd ("BMJ") its licensees and where the relevant Journal is co-owned by BMJ to the co-owners of the Journal, to publish the Work in this journal and any other BMJ products and to exploit all rights, as set out in our licence.

The Submitting Author accepts and understands that any supply made under these terms is made by BMJ to the Submitting Author unless you are acting as an employee on behalf of your employer or a postgraduate student of an affiliated institution which is paying any applicable article publishing charge ("APC") for Open Access articles. Where the Submitting Author wishes to make the Work available on an Open Access basis (and intends to pay the relevant APC), the terms of reuse of such Open Access shall be governed by a Creative Commons licence – details of these licences and which Creative Commons licence will apply to this Work are set out in our licence referred to above.

Other than as permitted in any relevant BMJ Author's Self Archiving Policies, I confirm this Work has not been accepted for publication elsewhere, is not being considered for publication elsewhere and does not duplicate material already published. I confirm all authors consent to publication of this Work and authorise the granting of this licence.

For peer review only

Compression Hosiery to Avoid Post-Thrombotic Syndrome (CHAPS) Protocol for a Randomised Controlled Trial [ISRCTN73041168]

Ankur Thapar[1], Rebecca Lawton[1], Laura J Burgess[1], Joseph Shalhoub[1], Andrew Bradbury, Nicky Cullum, David Epstein, Manjit Gohel, Robert Horne, Beverley J Hunt, John Norrie, Alun H Davies[1].

Corresponding author:

Ankur Thapar
FRCS (Gen Surg), PhD, FHEA, PG Cert. Med. Ed.
Consultant Vascular Surgeon and Honorary Senior Lecturer
Mid and South Essex NHS Foundation Trust & Imperial College London
a.thapar09@imperial.ac.uk

Rebecca Lawton
BSc
Trial manager
Imperial College London
r.lawton@imperial.ac.uk

Laura Burgess
MSc
Trial manager
Imperial College London
l.burgess@imperial.ac.uk

Joseph Shalhoub
BSc MBBS FHEA PhD MEd FRCS FEBVS
Consultant Vascular Surgeon & Honorary Clinical Senior Lecturer
Imperial College Healthcare NHS Trust & Imperial College London
j.shalhoub@imperial.ac.uk

Andrew Bradbury
FRCSEd, MD, MBA

For peer review only

Sampson Gamgee Professor of Vascular Surgery
University of Birmingham
a.w.bradbury@bham.ac.uk

Nicky Cullum
DBE, PhD, RGN, FMedSci, FAAN
Professor of Nursing
University of Manchester
nicky.cullum@manchester.ac.uk

David Epstein
MSc, PhD
Associate Professor at the Department of Applied Economics
University of Granada, Spain
davidepstein@ugr.es

Manjit Gohel
MD, FRCS, FEBVS
Consultant Vascular Surgeon
Cambridge University Hospitals
manjit.gohel@addenbrookes.nhs.uk

Robert Horne
BSc MSc PhD FRPS
Professor of Behavioural Medicine
University College London
r.horne@ucl.ac.uk

Beverley J Hunt
OBE, FRCP, FRCPath
Professor of Thrombosis and Haemostasis
Guy's and St Thomas' NHS Foundation Trust, London
beverley.hunt@gstt.nhs.uk

For peer review only

John Norrie

MSc

Professor of Medical Statistics and Trial Methodology

University of Edinburgh

j.norrie@ed.ac.uk

Alun Huw Davies

MA, DM, DSc, FRCS (Gen Surg), FHEA, FEBVS, FACPh

Professor of Vascular Surgery

Imperial College London

a.h.davies@imperial.ac.uk

Funder: This work was supported by the National Institute for Health Research (NIHR), Health Technology Assessment Programme, project number 17/147/47. The views expressed in this publication are those of the authors and not necessarily those of the NHS, NIHR or the UK Department of Health and Social Care. The HTA Programme is funded by the NIHR, with contributions from the Chief Scientist Office in Scotland and National Institute for Social Care and Health Research in Wales and the Health and Social Care R&D Division, Public Health Agency in Northern Ireland.

Sponsor: Imperial College London

Ethical approval: UK research ethics committee approval reference 17/LO/1585.

Study dates: 1st May 2020 – 1st Feb 2023

Conflict of Interest: none declared

Word count: 1833 (main body) + 653 (references)

Figures: 1

Tables: 1

For peer review only

For peer review only - http://bmjopen.bmj.com/site/about/guidelines.xhtml

Keywords: post-thrombotic syndrome, graduated compression stockings, deep vein thrombosis, adherence, randomised controlled trial

Author contributions:

AT developed protocol and drafted manuscript
RL PPI section and drafted manuscript
LB PPI section and drafted manuscript
JS developed protocol and drafted manuscript
AB developed protocol and drafted manuscript
NC developed protocol and drafted manuscript
DE developed health economic section and drafted manuscript
MG developed protocol and drafted manuscript
RH developed adherence substudy and drafted manuscript
BJ developed protocol and drafted manuscript
JN drafted manuscript, calculated sample size calculation, constructed data analysis plan
AHD overall project co-ordinator and drafted manuscript

Declaration: This protocol has been prepared in accordance with the SPIRIT 2013 checklist.

* I, the Submitting Author has the right to grant and does grant on behalf of all authors of the Work (as defined in the below author licence), an exclusive licence and/or a non-exclusive licence for contributions from authors who are: i) UK Crown employees; ii) where BMJ has agreed a CC-BY licence shall apply, and/or iii) in accordance with the terms applicable for US Federal Government officers or employees acting as part of their official duties; on a worldwide, perpetual, irrevocable, royalty-free basis to BMJ Publishing Group Ltd ("BMJ") its licensees and where the relevant Journal is co-owned by BMJ to the co-owners of the Journal, to publish the Work in BMJ Open and any other BMJ products and to exploit all rights, as set out in our licence.

The Submitting Author accepts and understands that any supply made under these terms is made by BMJ to the Submitting Author unless you are acting as an employee on behalf of your employer or a postgraduate student of an affiliated institution which is paying any applicable article publishing charge ("APC") for Open Access articles. Where the Submitting

For peer review only

137 Author wishes to make the Work available on an Open Access basis (and intends to pay the
138 relevant APC), the terms of reuse of such Open Access shall be governed by a Creative
139 Commons licence – details of these licences and which Creative Commons licence will apply
140 to this Work are set out in our licence referred to above.

For peer review only

For peer review only - http://bmjopen.bmj.com/site/about/guidelines.xhtml

Abstract

Introduction

Up to 50% of patients develop post-thrombotic syndrome after an above knee deep vein thrombosis (DVT). The aim of the study was to determine the effect of graduated compression stockings in preventing post-thrombotic syndrome (PTS) after DVT.

Methods and analysis

Pragmatic, UK multi-centre randomised trial in adults with first above knee DVT. The standard of care arm is anticoagulation. The intervention arm will receive anticoagulation plus stockings (European class II, 23-32mmHg compression) worn for a median of 18 months. The primary endpoint is PTS using the Villalta score. Analysis of this will be through a time to event approach and cumulative incidence at median 6, 12 and 18 months. A one-year process evaluation will examine factors contributing to adherence to stockings.

Ethics and Dissemination

UK research ethics committee approval (reference 17/LO/1918). Dissemination though the charity Thrombosis UK, the Imperial College London website, peer reviewed publications and international conferences.

Registration details

ISRCTN registration number 73041168.

Strengths and limitations of this study

- Pragmatic multi-centre randomised trial that will inform international practice
- Stockings are a low cost, widely applicable, safe intervention across high and low resource healthcare systems
- Assessor blind design
- Examines behavioural factors effecting adherence with stockings
- No placebo stocking arm due to ease of breaking blinding

For peer review only

Introduction

Deep vein thrombosis (DVT) occurs in 1-2 per 1000 adults in the UK(1) and half will go on to develop lifelong disability from post-thrombotic syndrome (PTS)(2). PTS is defined as "chronic venous symptoms or signs secondary to deep vein thrombosis"(3) e.g. leg pain, oedema and skin changes, progressing in 5% to venous ulceration. The average age of patients developing PTS is 55 years, meaning that most patients are of working age(4). Individuals with PTS have difficulty walking and staying in employment, and have a level of disability comparable to chronic obstructive pulmonary disease(5). The pathophysiology of PTS is sustained venous hypertension from venous outflow obstruction and valvular incompetence(6).

The recent negative results of the ATTRACT trial have refocussed attention on the effectiveness of graduated compression stockings (GCS) in preventing PTS(7). The UK National Institute for Health and Care Excellence (NICE) and the American College of Chest Physicians recently withdrew their recommendations for the use of GCS in the prevention of PTS based on the results of the Canadian SOX trial(8)(9). However 2018 European guidelines still recommend stockings(10).

A recent systematic review examined existing RCTs in this area(11). Three RCTs inclusive of 1177 patients examined the use of GCS providing 30-40mmHg compression at the ankle versus either no stocking (12,13), or a placebo stocking (14). Follow up ranged from 2-5 years with a primary outcome measure of cumulative incidence of PTS. There was important clinical, methodological and statistical heterogeneity between trials (I^2=94%). Key clinical differences were variable inclusion of patients with chronic venous disease, variable baseline rates of PTS and differing anatomy of DVT. Key methodological differences were use of a placebo stocking versus a no stocking control group, and differing PTS scoring systems(15). Additionally, adherence varied between 56% to 93%.

The largest placebo controlled trial showed no difference in the outcome of PTS with the use of stockings. The other two assessor blind trials showed absolute risk reductions of 23% and 39% with the use of stockings. There appeared to be more benefit from the use of GCS in populations with a higher baseline risk of PTS(11).

For peer review only - http://bmjopen.bmj.com/site/about/guidelines.xhtml

Compression Hosiery to Avoid Post-Thrombotic Syndrome (CHAPS) is a multicentre, pragmatic, assessor blind, RCT of adults with a first above knee DVT, comparing the regular use of a stocking with no stocking in preventing PTS.

PTS, together with associated complications such as venous thromboembolism and ulceration, comprise a substantial economic burden on health systems, patients and society at large due to days lost to illness. The cost of 3 pairs of high-quality graduated compression stockings per year is around £150, but this expense may be offset to some extent by lower costs elsewhere. Under standard care, around 50% of DVT result in PTS (16). Given the high cost of treatment of PTS, especially venous ulcers and the impact on quality of life(16), the addition of GCS could be a cost-effective addition to standard treatment.

For peer review only

For peer review only - http://bmjopen.bmj.com/site/about/guidelines.xhtml

Methods and analysis

CHAPS is a multi-centre, pragmatic, assessor-blind superiority RCT. The trial will follow patients up for a median of 18 months (range 6 – 30 months). Please see Figure 1 for a CONSORT diagram.

Eligibility

Table 1 details inclusion and exclusion criteria. Peripheral arterial disease will be screened for using pedal pulse palpation, with ankle brachial pressure index where pedal pulses are absent or equivocal.

Recruitment

Recruitment will be from emergency departments, ambulatory care, DVT, vascular or haematology clinics in 11 UK hospitals (see www.ISCTRN.com for details), via the National Institute for Health Research (NIHR) Clinical Research Network and trial nurses. Recruitment of 864 participants is planned over 24 months from both academic and non-academic centres.

Study arms

1. Standard care

Anticoagulation for a minimum of 3 months (as per NICE recommendations(8)). The type and duration of anticoagulation beyond 3 months will be determined by local guidelines with the expectation that this will be a direct oral anticoagulant for the majority. A placebo stocking arm was not included because of ease of breaking blinding. GCS are not be recommended for treatment of acute leg pain(17).

2. Intervention arm

Anticoagulation plus a standardised below knee compression stocking (European class II, 23-32mmHg compression) worn during waking hours until the end of the trial, or until an alternative is required, e.g. compression bandaging for venous ulceration. Minor variations such as change in fabric, open or closed toe or thigh length stockings are permissible if they aid adherence.

A number of low-cost behavioural aids will be made available to patients in the intervention arm:

- Patient education video at baseline

For peer review only

For peer review only - http://bmjopen.bmj.com/site/about/guidelines.xhtml

- Patient and carer training session for stocking donning and doffing at 2 weeks
- Free provision of a donning aid if required
- SMS reminders to wear stockings
- Web forum to provide peer to peer support regarding tips for use
- Cotton stocking for warm weather use

Randomisation

Participants will undergo 1:1 web-based randomisation to either control or intervention arm via the Research Electronic Data Capture (REDCap) database hosted at the Edinburgh Clinical Trials Unit.

Primary endpoint

The primary outcome measure is PTS as assessed by the recommended Villalta score(18). The primary outcome will be recorded at 6, 12 and 18 months, with 5 or more points indicating PTS and 15 or more points or an ulcer indicating severe PTS.

Secondary endpoints

- Employment status
- Change in disease-specific (VEnous INsufficiency Epidemiological and Economic Study-Quality of Life [VEINES-QoL]) and generic (EuroQoL-5 dimensions [EQ5D]) quality of life from baseline
- Adherence to GCS
- Healthcare resource use
- Adverse events

Sample size

The sample size calculation for CHAPS was based on the cumulative incidence of PTS at 18 months in the recent SOX trial(4). A minimum clinically important difference of a 10% absolute risk reduction in PTS with GCS was proposed based on the investment in behavioural change required by patients. With 864 participants randomised 1:1, the study will have 90% power at a 5% level of significance using a test of binomial proportions to detect an absolute reduction in the incidence of PTS from 30% in the standard care arm to 20% in the intervention arm, allowing for 10% loss to follow up. This reduction would represent a number needed to treat of 10 to prevent one case of PTS.

For peer review only - http://bmjopen.bmj.com/site/about/guidelines.xhtml

For peer review only

Internal pilot study

An internal pilot study will follow a randomly selected group of 100 patients over the first 12 months, leading to a mixed-methods process evaluation of factors contributing to GCS adherence.

Adaptations of the Medication Adherence Report Scale (MARS)(19), Brief Illness Perception Questionnaire (B-IPQ)(20), Beliefs about Medications Questionnaire (BMQ)(21), Treatment Intrusiveness Questionnaire (TIQ)(22) and a novel Beliefs about Stocking Questionnaire (BSQ) will be given to participants at the 2 week, 6 and 12 month follow up assessments. Additionally an adapted version regarding stocking usage will be emailed monthly to participants in both arms to examine self-initiated crossover. Qualitative interviews from a purposive sample of 20 patients at 1 month and 7 months will be used to examine factors affecting GCS adherence in further depth.

Trial stopping criteria

A combination of self-reported adherence and stocking reordering behaviour will be used to adjudicate adherence at the end of the pilot. The criteria for adequate one year adherence is ≥70% of participants in the intervention arm wearing the stocking for ≥4 days per week, with a documented stocking reorder in the last 6 months. This is the remit of the trial steering committee. If this is achieved, the trial will continue into the main study. If this is not achieved, the trial will terminate and a process evaluation of factors influencing adherence will be reported.

Assessment of outcomes and of blinding

An independent researcher at each site will perform leg assessments blind to treatment allocation. Participants will remove their stockings on the night prior to their clinic visit. The following questionnaires will be administered at follow up visits: EQ5D, VEINES-QoL, Social Presence and Privacy Questionnaire (SPPQ), MARS. Employment status and healthcare resource use will also be collected.

For peer review only

Blinding will be evaluated by asking assessors which arm they believe the participant is in. Unblinding is permissible only if a stocking related significant adverse event is suspected.

Data monitoring

In line with NIHR recommendations, a Trial Steering Committee (TSC) and an independent Data Monitoring Committee (iDMC) have been appointed to oversee trial conduct. A Trial Manager together with the Trial Management Group will oversee trial progress. The study will be monitored periodically by trial monitors to assess the progress of the study, verify adherence to the protocol and Good Clinical Practice guidelines and to review the completeness, accuracy and consistency of the data.

Data analysis

The primary analysis will be an intention-to-treat analysis that does not adjust for adherence to stockings. This will be performed independently by the Edinburgh Clinical Trials Unit. This will tell us the treatment effect given the adherence in the trial, which is appropriate to gauge real-world effectiveness. The occurrence of PTS will be analysed in both a time-to-first event approach (since it is possible that the treatment effect may not only avert PTS, but possibly delay its onset) and through analysis of cumulative incidence at a median of 6, 12 and 18 months (as recommended by the International Society for Thrombosis and Haemostasis)(3). Pre-specified subgroups analyses including iliac vein involvement and body mass index >30kg/m^2. To determine the effect of optimum adherence (wearing a stocking for the trial duration for ≥4 days per week) on outcome, a secondary analysis will use Complier Average Causal Estimation (CACE) modelling through instrumental variable regression. The results of the process evaluation will report which behavioural components change participants knowledge, beliefs and intentions regarding stocking usage.

Health economic analysis

A within-trial analysis and a decision model will be constructed. In both cases, the main analyses will be performed from the perspective of the UK NHS and Personal Social Services at 2018/19 prices. Secondary analyses will be performed from a societal perspective. The results of the analyses will be presented as estimates of mean incremental costs, effects, and, incremental cost per quality-adjusted life year. Sensitivity analyses will be conducted to test the robustness of the results to alternative assumptions about model structure, assumptions

For peer review only

For peer review only - http://bmjopen.bmj.com/site/about/guidelines.xhtml

and input data. Probabilistic sensitivity analysis will be conducted using Monte-Carlo simulation.

Ethics and dissemination

The trial was granted ethical approval (National Research Ethics Service ref. 19/LO/1585). Safety reporting will be performed as per the written guidance in the trial protocol. Dissemination of results will be professionally through peer reviewed journals and international conferences and to a lay audience including participants through the Thrombosis UK website.

Data will be filed for 10 years as per local policy.

Patient and Public Involvement

Patients, their carers' and relatives were involved in a 3-stage consultation process during the trial development stage, incorporating NIHR INVOLVE methodology. This consisted of a series of semi-structured interviews, a survey run via Thrombosis UK, and review of the CHAPS research plan and lay summary. Responses and feedback were incorporated into the design and budget of CHAPS. The Imperial Vascular PPI group has contributed 4 patients and 2 members of the public will advise the steering committee for the duration of the trial.

For peer review only

For peer review only - http://bmjopen.bmj.com/site/about/guidelines.xhtml

Table 1: Eligibility criteria

Inclusion criteria	*Exclusion criteria*
Symptomatic presentation of first deep vein thrombosis, <2 weeks from diagnosis	Previously intolerant of or already wearing graduated compression stockings for more than 1 month
Imaging confirmed, lower limb deep vein thrombosis (popliteal, femoral, iliac or combination)	Contraindication to wearing graduated compression stockings or allergy to fabric
Ability to give informed consent	Life expectancy <2 years
Age 18 years or over	Ankle brachial pressure index <0.8 (measured when pedal pulses absent or equivocal to palpation)
	Bilateral deep vein thrombosis
	Previous chronic venous insufficiency (patients with existing chronic skin changes or ulceration, defined as C4,5,6 by CEAP classification)
	Pre-existing post thrombotic syndrome, significant leg pain (e.g. knee arthritis, spinal claudication) or oedema (e.g. lymphoedema)
	Newly diagnosed cancer, metastatic cancer, or cancer undergoing active treatment or palliation
	Contraindication to anticoagulation

For peer review only

Figure 1: CONSORT diagram

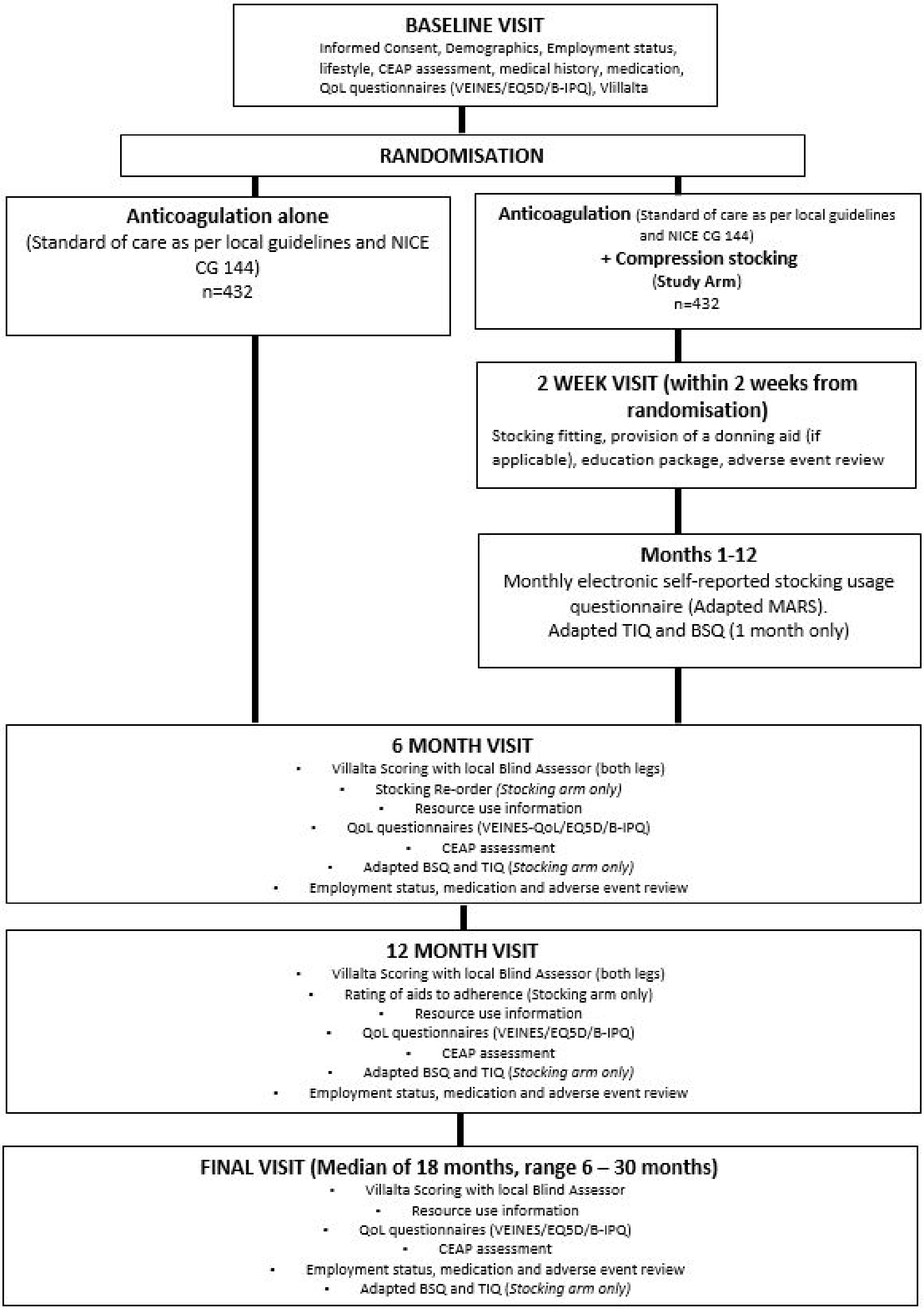

For peer review only - http://bmjopen.bmj.com/site/about/guidelines.xhtml

References

1. Raskob GE, Angchaisuksiri P, Blanco AN, Buller H, Gallus A, Hunt BJ, et al. Thrombosis: A major contributor to global disease burden. J Thromb Haemost. 2014;34(12):1580–90.
2. Kahn SR, Shrier I, Julian JA, Ducruet T, Arsenault L, Miron M-J, et al. Determinants and Time Course of the Postthrombotic Syndrome after Acute Deep Venous Thrombosis. Ann Intern Med. 2004;149(10):698–707.
3. Kahn SR, Partsch H, Vedantham S, Prandoni P, Kearon C. Definition of post-thrombotic syndrome of the leg for use in clinical investigations: A recommendation for standardization. J Thromb Haemost. 2009;7(5):879–83.
4. Kahn SR, Shapiro S, Wells PS, Rodger MA, Kovacs MJ, Anderson DR, et al. Compression stockings to prevent post-thrombotic syndrome: A randomised placebo-controlled trial. Lancet. 2014;383(9920):880–8.
5. Kahn SR, Shbaklo H, Lamping DL, Holcroft CA, Shrier I, Miron MJ, et al. Determinants of health-related quality of life during the 2 years following deep vein thrombosis. J Thromb Haemost. 2008;6(7):1105–12.
6. Kahn SR. The post-thrombotic syndrome: current knowledge, controversies, and directions for future research. Blood Rev. 2002;16:155–65.
7. Vedantham S, Magnuson E, Razavi MK, Comerota AJ, Gornik HL, Murphy TP, et al. Pharmacomechanical Catheter-Directed Thrombolysis for Deep-Vein Thrombosis. NEJM. 2017;377(23):2240–52.
8. NICE. National Institute for Health and Care Excellence. Venous thromboembolic diseases : CG144. 2018.
9. Kearon C, Akl EA, Ornelas J, Blaivas A, Jimenez D, Bounameaux H, et al. Antithrombotic therapy for VTE disease: CHEST guideline and expert panel report. Chest. 2016;149(2):315–52.
10. Rabe E, Partsch H, Hafner J, Lattimer C, Mosti G, Neumann M, et al. Indications for medical compression stockings in venous and lymphatic disorders : An evidence-based consensus statement. 2018;33(3):163–84.
11. Skervin AL, Thapar A, Franchini AJ, Prandoni P, Shalhoub J, Davies AH. Systematic Review and Meta-Analysis of Utility of Graduated Compression Stockings in

For peer review only - http://bmjopen.bmj.com/site/about/guidelines.xhtml

Prevention of Post-Thrombotic Syndrome. Eur J Vasc Endovasc Surg. 2016;51(6):838–45.

12. Prandoni P. Below-Knee Elastic Compression Stockings To Prevent the Post-Thrombotic Syndrome. Ann Intern Med. 2004;141(4):249–57.

13. Brandjes DP, Büller HR, Heijboer H, Huisman M V, de Rijk M, Jagt H, et al. Randomised trial of effect of compression stockings in patients with symptomatic proximal-vein thrombosis. Lancet. 1997 Mar;349(9054):759–62.

14. Kahn SR, Shapiro S, Wells PS, Rodger M a, Kovacs MJ, Anderson DR, et al. Compression stockings to prevent post-thrombotic syndrome: a randomised placebo-controlled trial. Lancet. 2014 Mar;383(9920):880–8.

15. Soosainathan A, Moore HM, Gohel MS, Davies AH. Scoring systems for the post-thrombotic syndrome. J Vasc Surg. 2013;57(1):254–61.

16. Epstein D, Gohel M, Heatley F, Davies AH. Cost-effectiveness of treatments for superficial venous reflux in patients with chronic venous ulceration. BJS Open. 2018;2(4):203–12.

17. Kahn SR, Shapiro S, Ducruet T, Wells PS, Rodger MA, Kovacs MJ, et al. Graduated compression stockings to treat acute leg pain associated with proximal DVT: A randomised controlled trial. Thromb Haemost. 2014;112(6):1137–41.

18. Kahn SR, Comerota AJ, Cushman M, Evans NS, Ginsberg JS, Goldenberg NA, et al. The postthrombotic syndrome: Evidence-based prevention, diagnosis, and treatment strategies: A scientific statement from the American heart association. Circulation. 2014;130(18):1636–61.

19. Thompson K, Kulkarni J, Sergejew AA. Reliability and validity of a new Medication Adherence Rating Scale (MARS) for the psychoses. Schizophr Res. 2000;42:241–7.

20. Broadbent E, Petrie KJ, Main J, Weinman J. The Brief Illness Perception Questionnaire. J Psychosom Res. 2006;60:631–7.

21. Horne R, Weinman J, Hankins M, Horne R, Weinman J, Hankins M. The beliefs about medicines questionnaire : The development and evaluation of a new method for assessing the cognitive representation of medication. Psychol Heal. 1999;14:1–24.

22. Cooper V, Horne R, Gellaitry G, Hons BA, Vrijens B, Lange A, et al. The Impact of Once-Nightly Versus Twice-Daily Dosing and Baseline Beliefs About HAART on Adherence to Efavirenz-Based HAART Over 48 Weeks : The NOCTE Study. J AIDS. 2010;53(3):369–

For peer review only

77.

For peer review only

For peer review only - http://bmjopen.bmj.com/site/about/guidelines.xhtml

STANDARD PROTOCOL ITEMS: RECOMMENDATIONS FOR INTERVENTIONAL TRIALS

SPIRIT 2013 Checklist: Recommended items to address in a clinical trial protocol and related documents*

Section/item	Item No	Description
**Administrative information**		
Title	1	Descriptive title identifying the study design, population, interventions, and, if applicable, trial acronym
Trial registration	2a	Trial identifier and registry name. If not yet registered, name of intended registry
	2b	All items from the World Health Organization Trial Registration Data Set
Protocol version	3	Date and version identifier
Funding	4	Sources and types of financial, material, and other support
Roles and responsibilities	5a	Names, affiliations, and roles of protocol contributors
	5b	Name and contact information for the trial sponsor
	5c	Role of study sponsor and funders, if any, in study design; collection, management, analysis, and interpretation of data; writing of the report; and the decision to submit the report for publication, including whether they will have ultimate authority over any of these activities
	5d	Composition, roles, and responsibilities of the coordinating centre, steering committee, endpoint adjudication committee, data management team, and other individuals or groups overseeing the trial, if applicable (see Item 21a for data monitoring committee)
**Introduction**		
Background and rationale	6a	Description of research question and justification for undertaking the trial, including summary of relevant studies (published and unpublished) examining benefits and harms for each intervention
	6b	Explanation for choice of comparators
Objectives	7	Specific objectives or hypotheses
Trial design	8	Description of trial design including type of trial (eg, parallel group, crossover, factorial, single group), allocation ratio, and framework (eg, superiority, equivalence, noninferiority, exploratory)

For peer review only

Methods: Participants, interventions, and outcomes

Study setting	9	Description of study settings (eg, community clinic, academic hospital) and list of countries where data will be collected. Reference to where list of study sites can be obtained
Eligibility criteria	10	Inclusion and exclusion criteria for participants. If applicable, eligibility criteria for study centres and individuals who will perform the interventions (eg, surgeons, psychotherapists)
Interventions	11a	Interventions for each group with sufficient detail to allow replication, including how and when they will be administered
	11b	Criteria for discontinuing or modifying allocated interventions for a given trial participant (eg, drug dose change in response to harms, participant request, or improving/worsening disease)
	11c	Strategies to improve adherence to intervention protocols, and any procedures for monitoring adherence (eg, drug tablet return, laboratory tests)
	11d	Relevant concomitant care and interventions that are permitted or prohibited during the trial
Outcomes	12	Primary, secondary, and other outcomes, including the specific measurement variable (eg, systolic blood pressure), analysis metric (eg, change from baseline, final value, time to event), method of aggregation (eg, median, proportion), and time point for each outcome. Explanation of the clinical relevance of chosen efficacy and harm outcomes is strongly recommended
Participant timeline	13	Time schedule of enrolment, interventions (including any run-ins and washouts), assessments, and visits for participants. A schematic diagram is highly recommended (see Figure)
Sample size	14	Estimated number of participants needed to achieve study objectives and how it was determined, including clinical and statistical assumptions supporting any sample size calculations
Recruitment	15	Strategies for achieving adequate participant enrolment to reach target sample size

Methods: Assignment of interventions (for controlled trials)

Allocation:

Sequence generation	16a	Method of generating the allocation sequence (eg, computer-generated random numbers), and list of any factors for stratification. To reduce predictability of a random sequence, details of any planned restriction (eg, blocking) should be provided in a separate document that is unavailable to those who enrol participants or assign interventions

For peer review only - http://bmjopen.bmj.com/site/about/guidelines.xhtml

Allocation concealment mechanism	16b	Mechanism of implementing the allocation sequence (eg, central telephone; sequentially numbered, opaque, sealed envelopes), describing any steps to conceal the sequence until interventions are assigned
Implementation	16c	Who will generate the allocation sequence, who will enrol participants, and who will assign participants to interventions
Blinding (masking)	17a	Who will be blinded after assignment to interventions (eg, trial participants, care providers, outcome assessors, data analysts), and how
	17b	If blinded, circumstances under which unblinding is permissible, and procedure for revealing a participant's allocated intervention during the trial
**Methods: Data collection, management, and analysis**		
Data collection methods	18a	Plans for assessment and collection of outcome, baseline, and other trial data, including any related processes to promote data quality (eg, duplicate measurements, training of assessors) and a description of study instruments (eg, questionnaires, laboratory tests) along with their reliability and validity, if known. Reference to where data collection forms can be found, if not in the protocol
	18b	Plans to promote participant retention and complete follow-up, including list of any outcome data to be collected for participants who discontinue or deviate from intervention protocols
Data management	19	Plans for data entry, coding, security, and storage, including any related processes to promote data quality (eg, double data entry; range checks for data values). Reference to where details of data management procedures can be found, if not in the protocol
Statistical methods	20a	Statistical methods for analysing primary and secondary outcomes. Reference to where other details of the statistical analysis plan can be found, if not in the protocol
	20b	Methods for any additional analyses (eg, subgroup and adjusted analyses)
	20c	Definition of analysis population relating to protocol non-adherence (eg, as randomised analysis), and any statistical methods to handle missing data (eg, multiple imputation)
**Methods: Monitoring**		
Data monitoring	21a	Composition of data monitoring committee (DMC); summary of its role and reporting structure; statement of whether it is independent from the sponsor and competing interests; and reference to where further details about its charter can be found, if not in the protocol. Alternatively, an explanation of why a DMC is not needed

	21b	Description of any interim analyses and stopping guidelines, including who will have access to these interim results and make the final decision to terminate the trial
Harms	22	Plans for collecting, assessing, reporting, and managing solicited and spontaneously reported adverse events and other unintended effects of trial interventions or trial conduct
Auditing	23	Frequency and procedures for auditing trial conduct, if any, and whether the process will be independent from investigators and the sponsor
**Ethics and dissemination**		
Research ethics approval	24	Plans for seeking research ethics committee/institutional review board (REC/IRB) approval
Protocol amendments	25	Plans for communicating important protocol modifications (eg, changes to eligibility criteria, outcomes, analyses) to relevant parties (eg, investigators, REC/IRBs, trial participants, trial registries, journals, regulators)
Consent or assent	26a	Who will obtain informed consent or assent from potential trial participants or authorised surrogates, and how (see Item 32)
	26b	Additional consent provisions for collection and use of participant data and biological specimens in ancillary studies, if applicable
Confidentiality	27	How personal information about potential and enrolled participants will be collected, shared, and maintained in order to protect confidentiality before, during, and after the trial
Declaration of interests	28	Financial and other competing interests for principal investigators for the overall trial and each study site
Access to data	29	Statement of who will have access to the final trial dataset, and disclosure of contractual agreements that limit such access for investigators
Ancillary and post-trial care	30	Provisions, if any, for ancillary and post-trial care, and for compensation to those who suffer harm from trial participation
Dissemination policy	31a	Plans for investigators and sponsor to communicate trial results to participants, healthcare professionals, the public, and other relevant groups (eg, via publication, reporting in results databases, or other data sharing arrangements), including any publication restrictions
	31b	Authorship eligibility guidelines and any intended use of professional writers
	31c	Plans, if any, for granting public access to the full protocol, participant-level dataset, and statistical code

For peer review only

Appendices

Informed consent materials	32	Model consent form and other related documentation given to participants and authorised surrogates
Biological specimens	33	Plans for collection, laboratory evaluation, and storage of biological specimens for genetic or molecular analysis in the current trial and for future use in ancillary studies, if applicable

*It is strongly recommended that this checklist be read in conjunction with the SPIRIT 2013 Explanation & Elaboration for important clarification on the items. Amendments to the protocol should be tracked and dated. The SPIRIT checklist is copyrighted by the SPIRIT Group under the Creative Commons "Attribution-NonCommercial-NoDerivs 3.0 Unported" license.

For peer review only

BMJ Open

Compression Hosiery to Avoid Post-Thrombotic Syndrome (CHAPS) Protocol for a Randomised Controlled Trial [ISRCTN73041168]

Journal:	*BMJ Open*
Manuscript ID	bmjopen-2020-044285.R1
Article Type:	Protocol
Date Submitted by the Author:	16-Feb-2021
Complete List of Authors:	Thapar, Ankur; Imperial College London, Academic Section of Vascular Surgery<br>Lawton, Rebecca; Imperial College London, Academic Section of Vascular Surgery<br>Burgess, Laura; Imperial College London, Academic Section of Vascular Surgery<br>Shalhoub, Joseph; Imperial College London, Section of Vascular Surgery<br>Bradbury, Andrew; University of Birmingham, Institute of Cardiovascular Sciences<br>Cullum, Nicky; The University of Manchester, School of Nursing, Midwifery & Social Work<br>Epstein, David; University of Granada, Faculty of Economic and Business Sciences<br>Gohel, Manjit; Addenbrooke's Hospital, Department of Vascular Surgery<br>Horne, Robert; University College London<br>Hunt, Beverley; Guy’s & St Thomas’ Foundation Trust, Dept of Haematology<br>Norrie, John; Edinburgh Clinical Trials Unit, University of Edinburgh No. 9, Bioquarter<br>Davies, AH; Imperial College London, Vascular
**Primary Subject Heading**:	Cardiovascular medicine
Secondary Subject Heading:	Emergency medicine, Haematology (incl blood transfusion)
Keywords:	Vascular medicine < INTERNAL MEDICINE, Anticoagulation < HAEMATOLOGY, Thromboembolism < CARDIOLOGY

SCHOLARONE™ Manuscripts

I, the Submitting Author has the right to grant and does grant on behalf of all authors of the Work (as defined in the below author licence), an exclusive licence and/or a non-exclusive licence for contributions from authors who are: i) UK Crown employees; ii) where BMJ has agreed a CC-BY licence shall apply, and/or iii) in accordance with the terms applicable for US Federal Government officers or employees acting as part of their official duties; on a worldwide, perpetual, irrevocable, royalty-free basis to BMJ Publishing Group Ltd ("BMJ") its licensees and where the relevant Journal is co-owned by BMJ to the co-owners of the Journal, to publish the Work in this journal and any other BMJ products and to exploit all rights, as set out in our licence.

The Submitting Author accepts and understands that any supply made under these terms is made by BMJ to the Submitting Author unless you are acting as an employee on behalf of your employer or a postgraduate student of an affiliated institution which is paying any applicable article publishing charge ("APC") for Open Access articles. Where the Submitting Author wishes to make the Work available on an Open Access basis (and intends to pay the relevant APC), the terms of reuse of such Open Access shall be governed by a Creative Commons licence – details of these licences and which Creative Commons licence will apply to this Work are set out in our licence referred to above.

Other than as permitted in any relevant BMJ Author's Self Archiving Policies, I confirm this Work has not been accepted for publication elsewhere, is not being considered for publication elsewhere and does not duplicate material already published. I confirm all authors consent to publication of this Work and authorise the granting of this licence.

For peer review only

Compression Hosiery to Avoid Post-Thrombotic Syndrome (CHAPS) Protocol for a Randomised Controlled Trial [ISRCTN73041168]

Ankur Thapar[1], Rebecca Lawton[1], Laura J Burgess[1], Joseph Shalhoub[1], Andrew Bradbury, Nicky Cullum, David Epstein, Manjit Gohel, Robert Horne, Beverley J Hunt, John Norrie, Alun H Davies[1].

Corresponding author:

Ankur Thapar

FRCS (Gen Surg), PhD, FHEA, PGCE, FEBVS

Consultant Vascular Surgeon and Honorary Senior Lecturer

Mid and South Essex NHS Foundation Trust, Imperial College London and Anglia Ruskin University

a.thapar09@imperial.ac.uk

Rebecca Lawton

BSc

Trial manager

Imperial College London

r.lawton@imperial.ac.uk

Laura Burgess

MSc

Trial manager

Imperial College London

l.burgess@imperial.ac.uk

Joseph Shalhoub

BSc MBBS FHEA PhD MEd FRCS FEBVS

Consultant Vascular Surgeon & Honorary Clinical Senior Lecturer

Imperial College Healthcare NHS Trust & Imperial College London

j.shalhoub@imperial.ac.uk

Andrew Bradbury

For peer review only

FRCSEd, MD, MBA
Sampson Gamgee Professor of Vascular Surgery
University of Birmingham
a.w.bradbury@bham.ac.uk

Nicky Cullum
DBE, PhD, RGN, FMedSci, FAAN
Professor of Nursing
University of Manchester
nicky.cullum@manchester.ac.uk

David Epstein
MSc, PhD
Associate Professor at the Department of Applied Economics
University of Granada, Spain
davidepstein@ugr.es

Manjit Gohel
MD, FRCS, FEBVS
Consultant Vascular Surgeon
Cambridge University Hospitals
manjit.gohel@addenbrookes.nhs.uk

Robert Horne
BSc MSc PhD FRPS
Professor of Behavioural Medicine
University College London
r.horne@ucl.ac.uk

Beverley J Hunt
OBE, FRCP, FRCPath
Professor of Thrombosis and Haemostasis
Guy's and St Thomas' NHS Foundation Trust, London
beverley.hunt@gstt.nhs.uk

For peer review only

John Norrie
MSc
Professor of Medical Statistics and Trial Methodology
University of Edinburgh
j.norrie@ed.ac.uk

Alun Huw Davies
MA, DM, DSc, FRCS (Gen Surg), FHEA, FEBVS, FACPh
Professor of Vascular Surgery
Imperial College London
a.h.davies@imperial.ac.uk

Protocol version: 5.0 (5/10/2020)

Funder: This work was supported by the National Institute for Health Research (NIHR), Health Technology Assessment Programme, project number 17/147/47. The views expressed in this publication are those of the authors and not necessarily those of the NHS, NIHR or the UK Department of Health and Social Care. The HTA Programme is funded by the NIHR, with contributions from the Chief Scientist Office in Scotland and National Institute for Social Care and Health Research in Wales and the Health and Social Care R&D Division, Public Health Agency in Northern Ireland.

Sponsor: Imperial College London

Declaration: The funder (NIHR) has been involved in study design and supports a clinical research network for recruitment. The sponsor is not been involved in study design or recruitment.

Ethical approval: UK research ethics committee approval reference 17/LO/1585.

Study dates: 1st May 2019 – 31st January 2023

Conflict of Interest: none declared

For peer review only - http://bmjopen.bmj.com/site/about/guidelines.xhtml

Word count: 2232 (main body) + 705 (references)

Figures: 1

Tables: 1

Appendices 4: (1) SPIRIT checklist, (2) consent form, (3) committees, (4) full protocol

Keywords: post-thrombotic syndrome, graduated compression stockings, deep vein thrombosis, adherence, randomised controlled trial

For peer review only

Author contributions:

AT developed protocol and drafted manuscript
RL PPI section and drafted manuscript
LB PPI section and drafted manuscript
JS developed protocol and drafted manuscript
AB developed protocol and drafted manuscript
NC developed protocol and drafted manuscript
DE developed health economic section and drafted manuscript
MG developed protocol and drafted manuscript
RH developed adherence substudy and drafted manuscript
BJ developed protocol and drafted manuscript
JN drafted manuscript, calculated sample size calculation, constructed data analysis plan
AHD overall project co-ordinator and drafted manuscript

Declaration: This protocol has been prepared in accordance with the SPIRIT 2013 checklist.

* I, the Submitting Author has the right to grant and does grant on behalf of all authors of the Work (as defined in the below author licence), an exclusive licence and/or a non-exclusive licence for contributions from authors who are: i) UK Crown employees; ii) where BMJ has agreed a CC-BY licence shall apply, and/or iii) in accordance with the terms applicable for US Federal Government officers or employees acting as part of their official duties; on a

For peer review only - http://bmjopen.bmj.com/site/about/guidelines.xhtml

worldwide, perpetual, irrevocable, royalty-free basis to BMJ Publishing Group Ltd ("BMJ") its licensees and where the relevant Journal is co-owned by BMJ to the co-owners of the Journal, to publish the Work in BMJ Open and any other BMJ products and to exploit all rights, as set out in our licence.

The Submitting Author accepts and understands that any supply made under these terms is made by BMJ to the Submitting Author unless you are acting as an employee on behalf of your employer or a postgraduate student of an affiliated institution which is paying any applicable article publishing charge ("APC") for Open Access articles. Where the Submitting Author wishes to make the Work available on an Open Access basis (and intends to pay the relevant APC), the terms of reuse of such Open Access shall be governed by a Creative Commons licence – details of these licences and which Creative Commons licence will apply to this Work are set out in our licence referred to above.

Data sharing statement: The datasets generated and/or analysed during this study will be included in the subsequent results publication.

For peer review only

Abstract

Introduction

Up to 50% of patients develop post-thrombotic syndrome after an above knee deep vein thrombosis (DVT). The aim of the study was to determine the effect of graduated compression stockings in preventing post-thrombotic syndrome (PTS) after DVT.

Methods and analysis

Pragmatic, UK multi-centre randomised trial in adults with first above knee DVT. The standard of care arm is anticoagulation. The intervention arm will receive anticoagulation plus stockings (European class II, 23-32mmHg compression) worn for a median of 18 months. The primary endpoint is PTS using the Villalta score. Analysis of this will be through a time to event approach and cumulative incidence at median 6, 12 and 18 months. An on-going process evaluation will examine factors contributing to adherence to stockings and to understand how and why the educational elements of the intervention were effective or ineffective.

Ethics and Dissemination

UK research ethics committee approval (reference 17/LO/1918). Dissemination though the charity Thrombosis UK, the Imperial College London website, peer reviewed publications and international conferences.

Registration details

ISRCTN registration number 73041168.

Strengths and limitations of this study

- Pragmatic multi-centre randomised trial that will inform international practice
- Stockings are a low cost, widely applicable, safe intervention across high and low resource healthcare systems
- Assessor blind design
- Examines behavioural factors effecting adherence with stockings
- No placebo stocking arm due to ease of breaking blinding

For peer review only

Introduction

Deep vein thrombosis (DVT) occurs in 1-2 per 1000 adults in the UK(1) and half will go on to develop lifelong disability from post-thrombotic syndrome (PTS)(2). PTS is defined as "chronic venous symptoms or signs secondary to deep vein thrombosis"(3) e.g. leg pain, oedema and skin changes, progressing in 5% to venous ulceration. The average age of patients developing PTS is 55 years, meaning that most patients are of working age(4). Individuals with PTS have difficulty walking and staying in employment, and have a level of disability comparable to chronic obstructive pulmonary disease(5). The pathophysiology of PTS is sustained venous hypertension from venous outflow obstruction and valvular incompetence(6).

The recent negative results of the ATTRACT trial have refocussed attention on the effectiveness of graduated compression stockings (GCS) in preventing PTS(7). The UK National Institute for Health and Care Excellence (NICE) and the American College of Chest Physicians recently withdrew their recommendations for the use of GCS in the prevention of PTS based on the results of the Canadian SOX trial(8)(9). However 2 European guidelines still recommend stockings(10)(11).

A recent systematic review examined existing RCTs in this area(12). Three RCTs inclusive of 1177 patients examined the use of GCS providing 30-40mmHg compression at the ankle versus either no stocking (13,14), or a placebo stocking (15). Follow up ranged from 2-5 years with a primary outcome measure of cumulative incidence of PTS. There was important clinical, methodological and statistical heterogeneity between trials (I^2=94%). Key clinical differences were variable inclusion of patients with chronic venous disease, variable baseline rates of PTS and differing anatomy of DVT. Key methodological differences were use of a placebo stocking versus a no stocking control group, and differing PTS scoring systems(16). Additionally, adherence varied between 56% to 93%.

The largest placebo-controlled trial showed no difference in the outcome of PTS with the use of stockings. The other two assessor blind trials showed absolute risk reductions of 23% and 39% with the use of stockings. There appeared to be more benefit from the use of GCS in populations with a higher baseline risk of PTS(12).

For peer review only

For peer review only - http://bmjopen.bmj.com/site/about/guidelines.xhtml

Compression Hosiery to Avoid Post-Thrombotic Syndrome (CHAPS) is a multicentre, pragmatic, assessor blind, RCT of adults with a first above knee DVT, comparing the regular use of a stocking with no stocking in preventing PTS.

PTS, together with associated complications such as venous thromboembolism and ulceration, comprise a substantial economic burden on health systems, patients and society at large due to days lost to illness. The cost of 3 pairs of high-quality graduated compression stockings per year is around £150, but this expense may be offset to some extent by lower costs elsewhere. Under standard care, around 50% of DVT result in PTS (16). Given the high cost of treatment of PTS, especially venous ulcers and the impact on quality of life(17), the addition of GCS could be a cost-effective addition to standard treatment.

For peer review only

Methods and analysis

CHAPS is a multi-centre, pragmatic, assessor-blind superiority RCT. The trial will follow patients up for a median of 18 months (range 6 – 30 months). The study commenced on 1st May 2019 and is due to close on 31st January 2023. Please see Figure 1 for a CONSORT diagram and Appendix 1 for a SPIRIT checklist.

Eligibility

Table 1 details inclusion and exclusion criteria. Peripheral arterial disease will be screened for using pedal pulse palpation, with ankle brachial pressure index where pedal pulses are absent or equivocal.

Recruitment

Recruitment will be from emergency departments, ambulatory care, DVT, vascular, obstetric or haematology clinics in 11 UK hospitals (see www.ISCTRN.com for details), via the National Institute for Health Research (NIHR) Clinical Research Network and trial nurses. Recruitment of 864 participants is planned over 24 months from both academic and non-academic centres. Informed consent will be obtained in writing (Appendix 2) by the local study nurse.

Study arms

1. <u>Standard care</u>

Anticoagulation for a minimum of 3 months (as per NICE recommendations(8)). The type and duration of anticoagulation beyond 3 months will be determined by local guidelines with the expectation that this will be a direct oral anticoagulant for the majority. A placebo stocking arm was not included because of ease of breaking blinding. GCS are not be recommended for treatment of acute leg pain(18).

2. <u>Intervention arm</u>

Anticoagulation plus a standardised below knee compression stocking (European class II, 23-32mmHg compression) worn during waking hours until the end of the trial, or until an alternative is required, e.g. compression bandaging for venous ulceration. Minor variations such as change in fabric, open or closed toe or thigh length stockings are permissible if they aid adherence.

For peer review only - http://bmjopen.bmj.com/site/about/guidelines.xhtml

For peer review only

A number of behavioural aids will be made available to patients in the stocking arm:

- Patient education video at baseline
- Patient and carer refresher session for stocking donning and doffing within 2 weeks
- Free provision of a donning aid if required
- Cotton stocking for summer use

The following participant retention strategies have been employed(19):

- Travel cost reimbursement
- Weekly text message reminders to wear stockings
- A Facebook support group for stocking wearers
- Next of kin contact for follow up

Stockings will be fitted and issued by a local research nurse at first visit. A donning aid will be supplied if required. Within 2 weeks there will be a face to face or video call refresher session for donning and doffing with the research nurse, patient and carer.

Randomisation

Participants will undergo 1:1 web-based randomisation by a local research nurse via the Research Electronic Data Capture (REDCap) database hosted at the Edinburgh Clinical Trials Unit.

Primary effectiveness endpoint

The primary outcome measure is PTS as assessed by the recommended Villalta score at a median of 18 months follow up(20). This will be supplemented by a time to onset of PTS model.

Secondary endpoints

1. Venous ulceration
2. Employment status (change in number of days working from baseline)
3. Quality of life measured using VEINES-QoL and EuroQoL EQ5D scales
4. Adherence to stockings and anticoagulants
5. Cost-effectiveness of stocking prescription

Sample size

For peer review only - http://bmjopen.bmj.com/site/about/guidelines.xhtml

For peer review only

The sample size calculation for CHAPS was based on the cumulative incidence of PTS at 18 months in the recent SOX trial(4). A minimum clinically important difference of a 10% absolute risk reduction in PTS with GCS was chosen multifactorially, based on patient consultation, the degree of behaviour change required by patients and to be less than that found in earlier positive stocking trials (ARR 23-39%)(13,14), reflecting improvements in anticoagulation. With 864 participants randomised 1:1, the study will have 90% power at a 5% level of significance using a test of binomial proportions to detect an absolute reduction in the incidence of PTS from 30% in the standard care arm to 20% in the intervention arm, allowing for 10% loss to follow up. This reduction would represent a number needed to treat of 10 to prevent one case of PTS.

Internal pilot study

An internal pilot study will follow a randomly selected group of 100 patients over the first 12 months, leading to a mixed-methods process evaluation of factors contributing to GCS adherence.

Adaptations of the Medication Adherence Report Scale (MARS)(21), Brief Illness Perception Questionnaire (B-IPQ)(22), Beliefs about Medications Questionnaire (BMQ)(23), Treatment Intrusiveness Questionnaire (TIQ)(24) and a novel Beliefs about Stocking Questionnaire (BSQ) will be given to participants at the 2 week, 6 and 12-month and final follow up assessments. Qualitative interviews from a purposive sample of 20 patients at 1 month and 7 months will be used to examine factors affecting GCS adherence in further depth.

Trial stopping criteria

A combination of self-reported adherence and stocking reordering behaviour will be used to adjudicate adherence at the end of the pilot. The criteria for adequate one year adherence is ≥70% of participants in the intervention arm wearing the stocking for ≥4 days per week, with a documented stocking reorder in the last 6 months. This is the remit of the trial steering committee. If this is achieved, the trial will continue into the main study. If this is not achieved, the trial will terminate and a process evaluation of factors influencing adherence will be reported.

For peer review only

Assessment of outcomes and of blinding

The study is assessor blind. An independent researcher at each site will perform Villalta assessments blind to treatment allocation. Participants will remove their stockings on the night prior to their clinic visit and be instructed not to discuss stockings during their assessment. The following questionnaires will be administered at follow up visits: EQ5D, VEINES-QoL and MARS. Employment status (average number of days per week currently working) and healthcare resource use (contacts and outcomes of interactions with health services) will also be collected. Data will be entered by the local research team onto the web-based database REDCap.

Blinding will be evaluated by asking assessors which arm they believe the participant is in. Unblinding is permissible only if a stocking related significant adverse event is suspected.

Data monitoring

In line with NIHR recommendations, a Trial Steering Committee (TSC) and an independent Data Monitoring Committee (iDMC) have been appointed to oversee trial conduct (please see Appendix 3). A Trial Manager together with the Trial Steering Committee will oversee trial progress. The study will be monitored by the Edinburgh Clinical Trials Unit to assess the progress of the study, verify adherence to the protocol and Good Clinical Practice guidelines and to review the completeness, accuracy and consistency of the data, through the use of independent data monitors. Pseudoanonymised data will be stored on REDCap, with a local key held by site Principal Investigators (PIs) to link this to clinical patient records. Data will be filed for 10 years as per local policy and then deleted.

For peer review only

Data analysis

The primary analysis will be an intention-to-treat analysis that does not adjust for adherence to stockings. This will be performed independently by the Edinburgh Clinical Trials Unit who will have sole access to the final REDCap dataset. This will determine the treatment effect given the adherence in the trial, which is appropriate to gauge real-world effectiveness. The occurrence of PTS will be analysed in both a time-to-PTS approach (since it is possible that the treatment effect may not only avert PTS, but possibly delay its onset) and through analysis of cumulative incidence at a median of 6, 12 and 18 months (as recommended by the International Society for Thrombosis and Haemostasis and peer reviewers)(3). Pre-specified subgroups analyses including iliac vein involvement and body mass index >30kg/m^2. To

determine the effect of optimum adherence (wearing a stocking for the trial duration for ≥4 days per week) on outcome, a secondary analysis will use Complier Average Causal Estimation (CACE) modelling through instrumental variable regression. The results of the process evaluation will report which behavioural components change participants knowledge, beliefs and intentions regarding stocking usage. Participants who discontinue the study will have information until date of leaving available for analysis.

Health economic analysis

Resource arising from the trial interventions, visits and admissions to hospital, general practice visits, community nursing and social and personal care will be collected during follow ups at 6 months, 12 months and the final visit and supplemented by case note review.

Employment status (average number of days worked per week, along with days lost from work and normal activities) will be collected from patients by questionnaire at baseline and at 6 months, 12 months and final follow-up.

A within-trial analysis and a decision model will be constructed. In both cases, the main analyses will be performed from the perspective of the UK NHS and Personal Social Services at 2018/19 prices. Secondary analyses will be performed from a societal perspective. The results of the analyses will be presented as estimates of mean incremental costs, effects, and, incremental cost per quality-adjusted life year. Sensitivity analyses will be conducted to test the robustness of the results to alternative assumptions about model structure, assumptions and input data. Probabilistic sensitivity analysis will be conducted using Monte-Carlo simulation.

Ethics and dissemination

The trial was granted ethical approval (National Research Ethics Service ref. 19/LO/1585). Protocol amendments will be circulated by email to investigators and study nurses to cascade to participants. Dissemination of results will be by the CHAPS co-investigators in peer reviewed journals and international conferences and to a lay audience through the Thrombosis UK website.

Adverse events and liability

For peer review only - http://bmjopen.bmj.com/site/about/guidelines.xhtml

All treatment related adverse events will be collected by site PIs. The chief investigator will be notified of all serious adverse events within 24 hours. All serious adverse events will be reported to the research ethics committee and sponsor, if, in the opinion of the chief investigator (CI), the event was related to the intervention. All related adverse events and serious adverse events will be recorded and summarised by treatment strategy. The sponsor (Imperial College London) holds a relevant insurance.

Patient and Public Involvement

Patients, their carers' and relatives were involved in a 3-stage consultation process during the trial development stage, incorporating NIHR INVOLVE methodology. This consisted of a series of semi-structured interviews, a survey run via Thrombosis UK, and review of the CHAPS research plan and lay summary. Responses and feedback were incorporated into the design and budget of CHAPS. The Imperial Vascular PPI group has contributed 4 patients and 2 members of the public will advise the steering committee for the duration of the trial.

For peer review only

For peer review only - http://bmjopen.bmj.com/site/about/guidelines.xhtml

Conclusion

The NIHR funded CHAPS trial will examine whether class II graduated compression stockings prevent post-thrombotic syndrome, are cost-effective and the factors influencing adherence at a median 18 months follow up (range 6-30 months).

For peer review only

For peer review only - http://bmjopen.bmj.com/site/about/guidelines.xhtml

Table 1: Eligibility criteria

Inclusion criteria	*Exclusion criteria*
Symptomatic presentation of first deep vein thrombosis, <2 weeks from diagnosis	Previously intolerant of or already wearing graduated compression stockings for more than 1 month
Imaging confirmed, lower limb deep vein thrombosis (popliteal, femoral, iliac or combination)	Contraindication to wearing graduated compression stockings or allergy to fabric
Ability to give informed consent	Life expectancy <2 years
Age 18 years or over	Ankle brachial pressure index <0.8 (measured when pedal pulses absent or equivocal to palpation)
	Bilateral deep vein thrombosis
	Previous chronic venous insufficiency (patients with existing chronic skin changes or ulceration, defined as C4,5,6 by CEAP classification)
	Pre-existing post thrombotic syndrome, significant leg pain (e.g. knee arthritis, spinal claudication) or oedema (e.g. lymphoedema)
	Newly diagnosed cancer, metastatic cancer, or cancer undergoing active treatment or palliation
	Contraindication to anticoagulation

Figure 1: CONSORT diagram

For peer review only

For peer review only - http://bmjopen.bmj.com/site/about/guidelines.xhtml

References

1. Raskob GE, Angchaisuksiri P, Blanco AN, Buller H, Gallus A, Hunt BJ, et al. Thrombosis: A major contributor to global disease burden. J Thromb Haemost. 2014;34(12):1580–90.
2. Kahn SR, Shrier I, Julian JA, Ducruet T, Arsenault L, Miron M-J, et al. Determinants and Time Course of the Postthrombotic Syndrome after Acute Deep Venous Thrombosis. Ann Intern Med. 2004;149(10):698–707.
3. Kahn SR, Partsch H, Vedantham S, Prandoni P, Kearon C. Definition of post-thrombotic syndrome of the leg for use in clinical investigations: A recommendation for standardization. J Thromb Haemost. 2009;7(5):879–83.
4. Kahn SR, Shapiro S, Wells PS, Rodger MA, Kovacs MJ, Anderson DR, et al. Compression stockings to prevent post-thrombotic syndrome: A randomised placebo-controlled trial. Lancet. 2014;383(9920):880–8.
5. Kahn SR, Shbaklo H, Lamping DL, Holcroft CA, Shrier I, Miron MJ, et al. Determinants of health-related quality of life during the 2 years following deep vein thrombosis. J Thromb Haemost. 2008;6(7):1105–12.
6. Kahn SR. The post-thrombotic syndrome: current knowledge, controversies, and directions for future research. Blood Rev. 2002;16:155–65.
7. Vedantham S, Magnuson E, Razavi MK, Comerota AJ, Gornik HL, Murphy TP, et al. Pharmacomechanical Catheter-Directed Thrombolysis for Deep-Vein Thrombosis. NEJM. 2017;377(23):2240–52.
8. NICE. National Institute for Health and Care Excellence. Venous thromboembolic diseases : CG144. 2018.
9. Kearon C, Akl EA, Ornelas J, Blaivas A, Jimenez D, Bounameaux H, et al. Antithrombotic therapy for VTE disease: CHEST guideline and expert panel report. Chest. 2016;149(2):315–52.
10. Rabe E, Partsch H, Hafner J, Lattimer C, Mosti G, Neumann M, et al. Indications for medical compression stockings in venous and lymphatic disorders : An evidence-based consensus statement. 2018;33(3):163–84.
11. Kakkos SK, Gohel M, Baekgaard N, Bauersachs R, Bellmunt-montoya S, Black SA, et al. European Society for Vascular Surgery (ESVS) 2021 Clinical Practice Guidelines on

For peer review only - http://bmjopen.bmj.com/site/about/guidelines.xhtml

the Management of Venous Thrombosis 5. Eur J Vasc Endovasc Surg [Internet]. 2021;in press. Available from: https://doi.org/10.1016/j.ejvs.2020.09.023

12. Skervin AL, Thapar A, Franchini AJ, Prandoni P, Shalhoub J, Davies AH. Systematic Review and Meta-Analysis of Utility of Graduated Compression Stockings in Prevention of Post-Thrombotic Syndrome. Eur J Vasc Endovasc Surg. 2016;51(6):838–45.

13. Prandoni P. Below-Knee Elastic Compression Stockings To Prevent the Post-Thrombotic Syndrome. Ann Intern Med. 2004;141(4):249–57.

14. Brandjes DP, Büller HR, Heijboer H, Huisman M V, de Rijk M, Jagt H, et al. Randomised trial of effect of compression stockings in patients with symptomatic proximal-vein thrombosis. Lancet. 1997 Mar;349(9054):759–62.

15. Kahn SR, Shapiro S, Wells PS, Rodger M a, Kovacs MJ, Anderson DR, et al. Compression stockings to prevent post-thrombotic syndrome: a randomised placebo-controlled trial. Lancet. 2014 Mar;383(9920):880–8.

16. Soosainathan A, Moore HM, Gohel MS, Davies AH. Scoring systems for the post-thrombotic syndrome. J Vasc Surg. 2013;57(1):254–61.

17. Epstein D, Gohel M, Heatley F, Davies AH. Cost-effectiveness of treatments for superficial venous reflux in patients with chronic venous ulceration. BJS Open. 2018;2(4):203–12.

18. Kahn SR, Shapiro S, Ducruet T, Wells PS, Rodger MA, Kovacs MJ, et al. Graduated compression stockings to treat acute leg pain associated with proximal DVT: A randomised controlled trial. Thromb Haemost. 2014;112(6):1137–41.

19. Teague S, Youssef GJ, Macdonald JA, Sciberras E, Shatte A, Fuller-tyszkiewicz M, et al. Retention strategies in longitudinal cohort studies : a systematic review and meta-analysis. BMC Med Res Meth. 2018;18(151):1–22.

20. Kahn SR, Comerota AJ, Cushman M, Evans NS, Ginsberg JS, Goldenberg NA, et al. The postthrombotic syndrome: Evidence-based prevention, diagnosis, and treatment strategies: A scientific statement from the American heart association. Circulation. 2014;130(18):1636–61.

21. Thompson K, Kulkarni J, Sergejew AA. Reliability and validity of a new Medication Adherence Rating Scale (MARS) for the psychoses. Schizophr Res. 2000;42:241–7.

22. Broadbent E, Petrie KJ, Main J, Weinman J. The Brief Illness Perception Questionnaire.

For peer review only

For peer review only - http://bmjopen.bmj.com/site/about/guidelines.xhtml

J Psychosom Res. 2006;60:631–7.

23. Horne R, Weinman J, Hankins M, Horne R, Weinman J, Hankins M. The beliefs about medicines questionnaire : The development and evaluation of a new method for assessing the cognitive representation of medication. Psychol Heal. 1999;14:1–24.

24. Cooper V, Horne R, Gellaitry G, Hons BA, Vrijens B, Lange A, et al. The Impact of Once-Nightly Versus Twice-Daily Dosing and Baseline Beliefs About HAART on Adherence to Efavirenz-Based HAART Over 48 Weeks : The NOCTE Study. J AIDS. 2010;53(3):369–77.

For peer review only

For peer review only - http://bmjopen.bmj.com/site/about/guidelines.xhtml

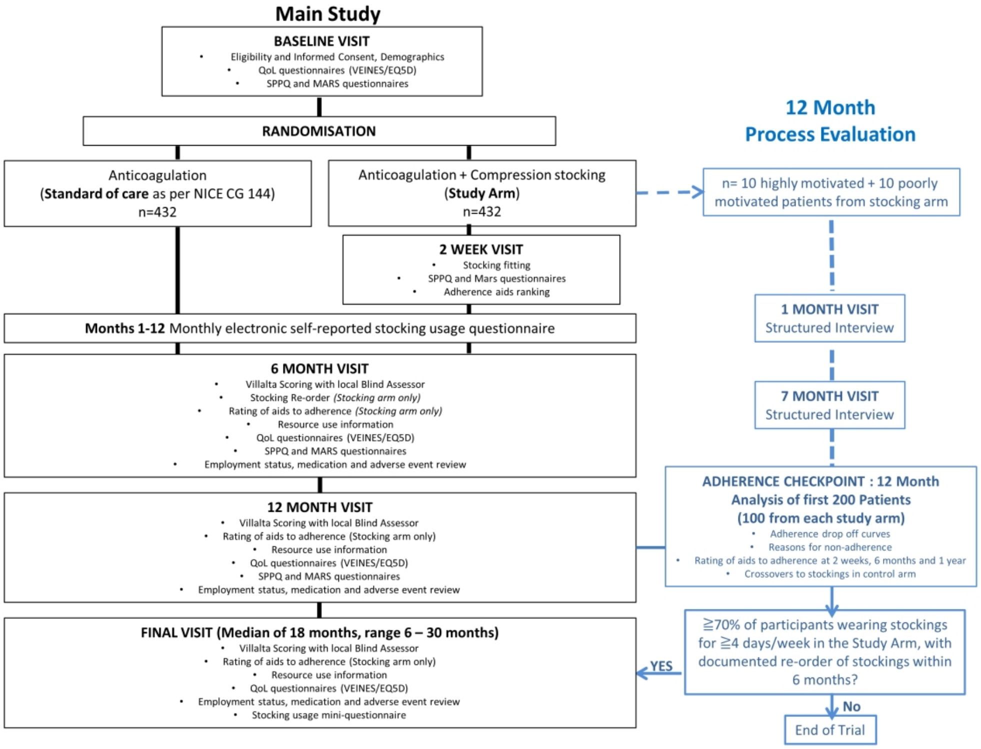

Figure 1: CONSORT diagram

254x190mm (300 x 300 DPI)

STANDARD PROTOCOL ITEMS: RECOMMENDATIONS FOR INTERVENTIONAL TRIALS

SPIRIT 2013 Checklist: Recommended items to address in a clinical trial protocol and related documents*

Section/item	Page	Item No	Description
**Administrative information**			
Title	1	1	Descriptive title identifying the study design, population, interventions, and, if applicable, trial acronym
Trial registration	1	2a	Trial identifier and registry name. If not yet registered, name of intended registry
		2b	All items from the World Health Organization Trial Registration Data Set
Protocol version	3	3	Date and version identifier
Funding	3	4	Sources and types of financial, material, and other support
Roles and responsibilities	4	5a	Names, affiliations, and roles of protocol contributors
	3	5b	Name and contact information for the trial sponsor
	3	5c	Role of study sponsor and funders, if any, in study design; collection, management, analysis, and interpretation of data; writing of the report; and the decision to submit the report for publication, including whether they will have ultimate authority over any of these activities
	App2	5d	Composition, roles, and responsibilities of the coordinating centre, steering committee, endpoint adjudication committee, data management team, and other individuals or groups overseeing the trial, if applicable (see Item 21a for data monitoring committee)
**Introduction**			
Background and rationale	7	6a	Description of research question and justification for undertaking the trial, including summary of relevant studies (published and unpublished) examining benefits and harms for each intervention
	9	6b	Explanation for choice of comparators
Objectives	10	7	Specific objectives or hypotheses
Trial design	9	8	Description of trial design including type of trial (eg, parallel group, crossover, factorial, single group), allocation ratio, and framework (eg, superiority, equivalence, noninferiority, exploratory)

For peer review only

For peer review only - http://bmjopen.bmj.com/site/about/guidelines.xhtml

Methods: Participants, interventions, and outcomes

Study setting	9	9	Description of study settings (eg, community clinic, academic hospital) and list of countries where data will be collected. Reference to where list of study sites can be obtained
Eligibility criteria	9	10	Inclusion and exclusion criteria for participants. If applicable, eligibility criteria for study centres and individuals who will perform the interventions (eg, surgeons, psychotherapists)
Interventions	9	11a	Interventions for each group with sufficient detail to allow replication, including how and when they will be administered
	9	11b	Criteria for discontinuing or modifying allocated interventions for a given trial participant (eg, drug dose change in response to harms, participant request, or improving/worsening disease)
	9	11c	Strategies to improve adherence to intervention protocols, and any procedures for monitoring adherence (eg, drug tablet return, laboratory tests)
	9	11d	Relevant concomitant care and interventions that are permitted or prohibited during the trial
Outcomes	10	12	Primary, secondary, and other outcomes, including the specific measurement variable (eg, systolic blood pressure), analysis metric (eg, change from baseline, final value, time to event), method of aggregation (eg, median, proportion), and time point for each outcome. Explanation of the clinical relevance of chosen efficacy and harm outcomes is strongly recommended
Participant timeline	15	13	Time schedule of enrolment, interventions (including any run-ins and washouts), assessments, and visits for participants. A schematic diagram is highly recommended (see Figure)
Sample size	10	14	Estimated number of participants needed to achieve study objectives and how it was determined, including clinical and statistical assumptions supporting any sample size calculations
Recruitment	9	15	Strategies for achieving adequate participant enrolment to reach target sample size

Methods: Assignment of interventions (for controlled trials)

Allocation:			
Sequence generation	10	16a	Method of generating the allocation sequence (eg, computer-generated random numbers), and list of any factors for stratification. To reduce predictability of a random sequence, details of any planned restriction (eg, blocking) should be provided in a separate document that is unavailable to those who enrol participants or assign interventions

Allocation concealment mechanism	10	16b	Mechanism of implementing the allocation sequence (eg, central telephone; sequentially numbered, opaque, sealed envelopes), describing any steps to conceal the sequence until interventions are assigned
Implementation	10	16c	Who will generate the allocation sequence, who will enrol participants, and who will assign participants to interventions
Blinding (masking)	11	17a	Who will be blinded after assignment to interventions (eg, trial participants, care providers, outcome assessors, data analysts), and how
	11	17b	If blinded, circumstances under which unblinding is permissible, and procedure for revealing a participant's allocated intervention during the trial
**Methods: Data collection, management, and analysis**			
Data collection methods	10,11	18a	Plans for assessment and collection of outcome, baseline, and other trial data, including any related processes to promote data quality (eg, duplicate measurements, training of assessors) and a description of study instruments (eg, questionnaires, laboratory tests) along with their reliability and validity, if known. Reference to where data collection forms can be found, if not in the protocol
	12	18b	Plans to promote participant retention and complete follow-up, including list of any outcome data to be collected for participants who discontinue or deviate from intervention protocols
Data management	12	19	Plans for data entry, coding, security, and storage, including any related processes to promote data quality (eg, double data entry; range checks for data values). Reference to where details of data management procedures can be found, if not in the protocol
Statistical methods	12	20a	Statistical methods for analysing primary and secondary outcomes. Reference to where other details of the statistical analysis plan can be found, if not in the protocol
	12	20b	Methods for any additional analyses (eg, subgroup and adjusted analyses)
	12	20c	Definition of analysis population relating to protocol non-adherence (eg, as randomised analysis), and any statistical methods to handle missing data (eg, multiple imputation)
**Methods: Monitoring**			
Data monitoring	App2	21a	Composition of data monitoring committee (DMC); summary of its role and reporting structure; statement of whether it is independent from the sponsor and competing interests; and reference to where further details about its charter can be found, if not in the protocol. Alternatively, an explanation of why a DMC is not needed

	11	21b	Description of any interim analyses and stopping guidelines, including who will have access to these interim results and make the final decision to terminate the trial
Harms	12	22	Plans for collecting, assessing, reporting, and managing solicited and spontaneously reported adverse events and other unintended effects of trial interventions or trial conduct
Auditing	12	23	Frequency and procedures for auditing trial conduct, if any, and whether the process will be independent from investigators and the sponsor
**Ethics and dissemination**			
Research ethics approval	13	24	Plans for seeking research ethics committee/institutional review board (REC/IRB) approval
Protocol amendments	13	25	Plans for communicating important protocol modifications (eg, changes to eligibility criteria, outcomes, analyses) to relevant parties (eg, investigators, REC/IRBs, trial participants, trial registries, journals, regulators)
Consent or assent	9	26a	Who will obtain informed consent or assent from potential trial participants or authorised surrogates, and how (see Item 32)
	9	26b	Additional consent provisions for collection and use of participant data and biological specimens in ancillary studies, if applicable
Confidentiality	12	27	How personal information about potential and enrolled participants will be collected, shared, and maintained in order to protect confidentiality before, during, and after the trial
Declaration of interests	3	28	Financial and other competing interests for principal investigators for the overall trial and each study site
Access to data	12	29	Statement of who will have access to the final trial dataset, and disclosure of contractual agreements that limit such access for investigators
Ancillary and post-trial care	13	30	Provisions, if any, for ancillary and post-trial care, and for compensation to those who suffer harm from trial participation
Dissemination policy	13	31a	Plans for investigators and sponsor to communicate trial results to participants, healthcare professionals, the public, and other relevant groups (eg, via publication, reporting in results databases, or other data sharing arrangements), including any publication restrictions
	13	31b	Authorship eligibility guidelines and any intended use of professional writers
	12	31c	Plans, if any, for granting public access to the full protocol, participant-level dataset, and statistical code

For peer review only - http://bmjopen.bmj.com/site/about/guidelines.xhtml

Appendices

Informed consent materials	App1	32	Model consent form and other related documentation given to participants and authorised surrogates
Biological specimens	n.a	33	Plans for collection, laboratory evaluation, and storage of biological specimens for genetic or molecular analysis in the current trial and for future use in ancillary studies, if applicable

*It is strongly recommended that this checklist be read in conjunction with the SPIRIT 2013 Explanation & Elaboration for important clarification on the items. Amendments to the protocol should be tracked and dated. The SPIRIT checklist is copyrighted by the SPIRIT Group under the Creative Commons "Attribution-NonCommercial-NoDerivs 3.0 Unported" license.

For peer review only

CHAPS Trial Commitees

Trial Steering Committee

Bold = independent member

**Dr**	**Peter**	**MacCallum**	**Senior Lecturer in Haematology**	**Chair**
**Dr**	**Susie**	**Shapiro**	**Consultant Haematologist**	**Member**
**Mr**	**Isaac**	**Nyameke**	**Consultant Vascular Surgeon**	**Member**
**Dr**	**Stephen**	**Gerry**	**Senior medical statistician and NIHR doctoral research fellow**	**Member**
Professor	Alun	Davies	Professor of Vascular Surgery	Member
**Mr**	**Andrew**	**Steptowe**	**PPI member**	**Public Observer**
**Mr**	**David**	**Brae**	**PPI member**	**Public Observer**
Mr	Ankur	Thapar	Co-applicant	Observer
Professor	John	Norrie	Senior Statistician	Observer

Data monitoring committee

Bold = independent member

**Mr**	**Richard**	**Bulbulia**	**Consultant Vascular Surgeon**	**Chair**
**Dr**	**Natalie**	**Staplin**	**Senior Statistician – Renal Studies Group**	**Member**
**Mr**	**Richard**	**Haynes**	**Associate Professor**	**Member**
**Mr**	**Keith**	**Poskitt**	**Consultant Vascular and General Surgeon**	**Member**
Mr	Imad	Adamestam	Trial Statistician	

For peer review only

Delete this line, then print on CHAPS Hospital/Trust headed pape

CHAPS

Compression Hosiery to Avoid Post-Thrombotic Syndrome

(CHAPS)

ISRCTN: 73041168

HRA/REC Reference: 19/LO/1585

IRAS 263041

Sponsor Reference: 19CX5434

For peer review only

Acknowledgement

This project is funded by a National Institute for Health Research, Health Technology Assessment programme grant, which is funded by the National Institute for Health Research (NIHR)

Disclaimer

The views expressed in this publication are those of the author(s) and not necessarily those of the MRC, NHS, NIHR or the Department of Health.

For peer review only - http://bmjopen.bmj.com/site/about/guidelines.xhtml

Delete this line, then print on Hospital/Trust headed paper

Site ID:	Initials:
Participant Trial ID:	Principal Investigator Name:

Compression Hosiery to Avoid Post-thrombotic Syndrome (CHAPS)

IRAS 263041

PATIENT CONSENT FORM

Please initial box

1. I confirm that I have read and understand the information sheet dated 25/06/2020 (Version 3.0) for the above study and have had the opportunity to ask questions which have been answered fully.

2. I understand that my participation is voluntary and that I am free to leave the study at any time without my medical care or legal rights being affected.

3. I understand that relevant sections of my medical records may be looked at by authorised individuals from the research team, from regulatory bodies, from the study Sponsor, or from the NHS Trust in order to check that the study is being carried out correctly. I give permission, provided that strict confidentiality is maintained, for these bodies to have access to my medical records for the above study.

4. I understand that my mobile phone number and email address will be stored until the end of the study securely by the University of Edinburgh and used to send weekly text message reminders to you until your participation on the study ends

5. I understand that my pseudonymised data will be transferred to the University Of Granada for the analysis.

6. I agree to my data being entered onto a secure database held at the University of Edinburgh, in accordance with the Data Protection Act 2018.

7. I agree to my GP, or any other doctor treating me, being notified of my participation in this study. I agree to my GP being involved in the study, including any necessary exchange of information about me between my GP and the research team.

For peer review only - http://bmjopen.bmj.com/site/about/guidelines.xhtml

8. If during the study my clinical care team determine that I have lost capacity to provide informed consent, I will be withdrawn from the study and any identifiable data collected with consent would be retained and used in the study. ☐

9. I agree to take part in the CHAPS study. ☐

Optional consent section (please initial the appropriate box)

10. I give/do not give consent for information collected about me to be used to support other research in the future, including those outside of the EEA.

Give consent ☐ Do not give consent ☐

11. I give/do not give consent for my data may be linked with appropriate national databases, including Hospital Episode Statistics (HES), and the National Vascular Database as well as for longer term follow-up in the event the trial is extended

Give consent ☐ Do not give consent ☐

12. I give/do not give consent to be contacted in the future with regards to this study, should the study be extended.

Give consent ☐ Do not give consent ☐

Full Name of Participant	Date	Signature
Name of Person Taking Consent	Date	Signature

(1 copy for participant; 1 copy for the patient's medical notes, 1 copy for the site file)

For peer review only - http://bmjopen.bmj.com/site/about/guidelines.xhtml
